# Predictive Factors of Non-Elevation of Carcinoembryonic Antigen 125 in Acute Heart Failure

**DOI:** 10.3390/life15030494

**Published:** 2025-03-18

**Authors:** Raquel López-Vilella, Francisco González-Vílchez, Borja Guerrero Cervera, Víctor Donoso Trenado, Zoser Saura Carretero, Julia Martínez-Solé, Sara Huélamo Montoro, Luis Martínez Dolz, Luis Almenar Bonet

**Affiliations:** 1 Cardiology Department, Hospital Universitari i Politècnic La Fe, Fernando Abril Martorell Avenue 106, 46026 Valencia, Spain; lopez_raqvil@gva.es (R.L.-V.); vdonoso@outlook.com (V.D.T.); zoser.saura@gmail.com (Z.S.C.); juliamsole@gmail.com (J.M.-S.); huelamo_sar@gva.es (S.H.M.); martinez_luidol@gva.es (L.M.D.); lualmenar@gmail.com (L.A.B.); 2 Heart Failure and Transplant Unit, Hospital Universitari i Politècnic La Fe, 46026 Valencia, Spain; 3 Cardiology Department, Hospital Universitario Marqués de Valdecilla, 39008 Santander, Spain; cargvf@gmail.com; 4 Centro de Investigación Biomédica en Red de Enfermedades Cardiovasculares (CIBERCV), Instituto de Salud Carlos III, 28029 Madrid, Spain

**Keywords:** carbohydrate antigen 125 (CA-125), low CA-125, acute heart failure, prognosis

## Abstract

This study aims to analyze the factors associated with the lack of carbohydrate antigen 125 (CA-125) elevation in cases of acute heart failure (HF) decompensation. This retrospective study was conducted on 3167 consecutive patients admitted for acute HF in the cardiology department of a referral hospital (June 2019 to June 2024). Admissions from outpatient clinics (n: 1018) and transfers from other hospitals (n: 752) were excluded. The variables of interest included clinical, echocardiographic, therapeutic, and analytical factors. Low CA-125 levels were defined as values ≤ 50 U/mL. A total of 1397 patients were included, of whom 515 had normal CA-125 levels and 882 had elevated levels. Clinically, independent predictors of low CA-125 were sinus rhythm on electrocardiogram (OR: 1.42, 95% CI: 1.12–1.64; *p*: 0.003) and sleep apnea–hyponpnea syndrome (OR: 1.76, 95% CI: 1.15–2.70; *p*: 0.009). Echocardiographically, inferior vena cava collapse greater than 50% with inspiration was associated with low CA-125 (OR: 1.78, 95% CI: 1.19–2.69; *p* = 0.005), as well as with non-severe right ventricular dysfunction. (OR: 2.42; IC95%: 1.39–4.20; *p*: 0.002). Analytically, elevated NT-proBNP levels were associated with elevated CA-125 levels (OR: 0.99; IC95%: 0.99–0.99; *p*: 0.006). Survival was higher in the group with CA-125 ≤ 50 U/mL (*p*: 0.019). Conversely, as CA-125 values increased, mortality also rose. In conclusion, the absence of CA-125 elevation in patients admitted for acute HF is associated with sinus rhythm, sleep apnea–hyponpnea syndrome, low NT-proBNP levels, and inferior vena cava collapse greater than 50% with inspiration.

## 1. Introduction

Biomarkers are biological molecules found in blood and other tissues that serve as indicators of pathological processes. In heart failure (HF), several biomarkers have been standardized due to their consistent elevation during episodes of decompensation, leading to hospital admission [1]. Among the most commonly used are C-reactive protein (CRP), utilized for inflammation; procalcitonin, utilized for infection; troponin T (TnT), utilized for myocardial necrosis; and, most notably, the amino-terminal fragment of B-type natriuretic peptide (NT-proBNP), utilized for myocardial stress [2,3,4]. Recent research has explored additional biomarkers that could improve HF management. One such candidate is carbohydrate antigen 125 (CA-125), a mucin primarily used in the diagnosis and follow-up of ovarian cancer, which has recently gained attention in the context of HF [1]. Elevated CA-125 levels have been associated with HF decompensation and congestion, suggesting a potential role in disease monitoring and prognosis [5,6]. The biological rationale behind CA-125 elevation in HF stems from its synthesis in coelomic epithelium, including mesothelial structures such as the pleura, peritoneum, and pericardium, as well as Müllerian-derived tissues (fallopian tubes, endocervix, and vaginal fundus). Given its high molecular weight, CA-125 is believed to rise in response to pleuropulmonary involvement in left HF decompensation and peritoneal involvement in right HF decompensation [7]. Consequently, higher levels have been correlated with increased congestion [6]. However, despite its potential utility, the role of CA-125 in HF remains controversial. While some patients with decompensated HF present with elevated CA-125 levels upon admission, others exhibit normal values despite severe clinical deterioration. This discrepancy suggests that multiple factors—including clinical presentation, laboratory findings, echocardiographic parameters, and pharmacological influences—may modulate CA-125 expression in HF [7]. The precise mechanisms underlying this variability remain unclear, highlighting the need for further investigation to determine CA-125’s prognostic and clinical value in HF management.

The hypothesis of this study was that many patients admitted for decompensated HF have CA-125 levels in normal range and that there would be clinical, analytical, exploratory and therapeutic factors associated with this fact. This knowledge would help to deepen our understanding of this new molecular marker and its lack of response in certain cases, which could have important clinical implications. Thus, the main objective of this study was to analyze the causes associated with a lack of CA-125 elevation in acute cardiac decompensation leading to hospital admission. The secondary objectives were to assess the prognostic impact of this biological marker on survival during follow-up and to analyze the prediction of mortality risk according to the CA-125 value obtained on admission.

## 2. Materials and Methods

This is a retrospective study based on a database of patients consecutively admitted for an episode of decompensated HF in the cardiology department of a tertiary hospital. The information was collected during the patient’s admission and was extracted and stored by a team of clinical cardiologists with expertise in HF. Recruitment was consecutive over five years (June 2019–June 2024), including 3167 patients. This study aims to analyze patients with acute HF requiring urgent hospital admission. Therefore, patients referred from outpatient clinics were excluded (n = 1018). Patients transferred from other centers were also excluded to ensure a more homogeneous sample, as they had often spent several days in another facility and thus arrived at our center at a different stage of acute HF (n = 752). The final number of patients included in the study was 1397, with normal CA-125 in 515 patients and elevated CA-125 in 882 patients. CA-125 levels were measured at admission. CA-125 was considered normal up to 50 U/mL. The upper limit of normality in HF is not well defined. For suspected ovarian cancer, this threshold is generally set at 35 U/mL, although it depends on the laboratory [8].

The variables of interest included clinical parameters (history and baseline characteristics), echocardiographic findings (functional assessment of both ventricles and the inferior vena cava), therapeutic aspects (medications taken at the time of admission), and laboratory parameters [usual biomarkers in these patients, based on a preconfigured analytical profile specific to decompensated HF] [9].

This study was conducted in accordance with the Declaration of Helsinki. The research project was approved by the Biomedical Research Ethics Committee of Hospital Universitario y Politécnico La Fe de Valencia.

### Statistical Analysis

Categorical variables were expressed as percentages, while numerical variables were reported as mean (standard deviation) or median (interquartile range) values, depending on their distribution, which was assessed using the Kolmogorov–Smirnov test. Group comparisons were performed using the Chi-square test with Yates’ correction when appropriate for categorical variables, and either Student’s *t*-test or the Mann–Whitney *U* test for continuous variables, depending on the normality of their distribution. Missing data were handled using multiple imputation with chained equations. To ensure adequate convergence, we excluded variables with more than 50% missing data (platelet count, left atrial diameter, and serum troponin levels), variables with very low event counts (use of vericiguat), and closely related or redundant variables. Specifically, serum creatinine, glomerular filtration rate, potassium, and sodium were excluded due to their correlation with renal dysfunction, transaminase levels were excluded due to their correlation with bilirubin, transferrin saturation was excluded due to its correlation with ferritin, and hematocrit was excluded due to its correlation with hemoglobin. Additionally, significant left-sided valvular heart disease and non-loop diuretics were grouped into two distinct variables. Overall, missing data affected 5.99% of the dataset. A total of 15 imputed datasets containing 41 variables were generated. Categorical variables were imputed using logistic or multinomial regression, as appropriate, while continuous variables were imputed using linear regression. Regression results were pooled across imputed datasets following Rubin’s rules. Predictors of low CA125 levels were assessed using logistic regression, with CA125 dichotomized at 50 U/mL as the dependent variable and all 41 variables included as predictors. No variable selection methods were applied, as there were 12.6 cases of low CA125 per variable. Additionally, the prognostic impact of low CA125 levels was analyzed using Kaplan–Meier curves, and survival differences were assessed with the log-rank test. A *p*-value < 0.05 was considered statistically significant. Statistical analyses were performed using IBM SPSS Statistics version 27^®^ and Stata^®^ version 16.1. Graphs were generated using SPSS and PowerPoint.

## 3. Results

### 3.1. Antecedents and Clinical Congestive Profile

CA-125 levels are most frequently elevated in patients admitted for acute cardiac decompensation requiring hospitalization, with a ratio of 882/515 (1.7:1). In the univariate analysis, numerous significant differences were observed when comparing the baseline characteristics of both groups. Patients with low CA-125 levels on admission were more often older women with hypertension, displaying higher prevalence of sinus rhythm, obesity, and sleep apnea–hypopnea syndrome (SAHS) (Table 1).

**Figure 1:**
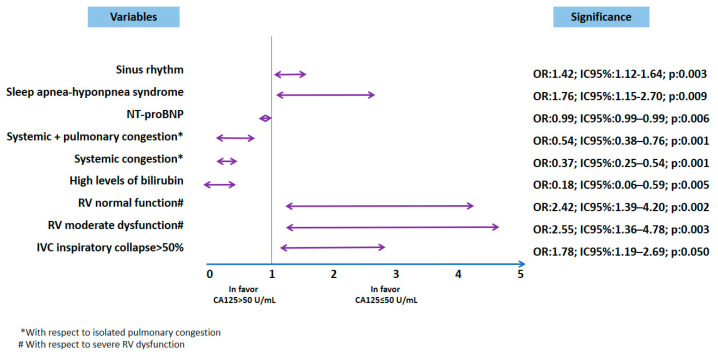
Multivariable analysis. AUC = 0.73 (0.70–0.76); *p* < 0.0001. Figure legend: * with respect to isolated pulmonary congestion. # regarding mild depression of RV function. Abbreviations: CA-125: carbohydrate antigen 125;; NT-proBNP: N-terminal pro-B-type natriuretic peptide; RV: right ventricle.

However, in the multivariable analysis, a significant association with CA-125 levels ≤ 50 U/mL on admission was found in patients with sinus rhythm on electrocardiogram and patients with SAHS (Figure 1). The variables analyzed included de novo HF, functional class (New York Heart Association, NYHA), congestion pattern, and a history of prior hospitalizations since diagnosis. Pulmonary congestion was more prevalent in the normal CA-125 group. Multivariable analysis demonstrated that all congestion patterns, compared to the pulmonary congestion pattern, were more strongly associated with elevated CA-125 levels (Table 2).

### 3.2. Admission Treatment and Laboratory Tests

Many patients were taking medication at the time of hospital admission. However, in the multivariable analysis, none of these medications were associated with low CA-125 levels. In the univariate comparison, there was a trend toward a lower percentage of patients taking mineralocorticoid receptor antagonists (MRAs) and sodium–glucose cotransporter 2 (SGLT2) inhibitors (Table 3). Significant differences were observed in admission blood tests between the two groups. Patients with low CA-125 levels had lower bilirubin, transaminase, and NT-proBNP levels. However, multivariable analysis identified a significant association with plasma NT-proBNP levels, showing that lower NT-proBNP values were associated with CA-125 levels ≤ 50 U/mL and bilirubin levels (Table 4 and Figure 1).

### 3.3. Ventricular Function Analysis

Patients with CA-125 ≤ 50 U/mL more frequently presented preserved left ventricular ejection fraction (LVEF), with normal left ventricular (LV) size and increased measurements of the interventricular septum and left atrium. Also, right ventricular (RV) function was normal in a higher percentage of patients, with less dilatation of this chamber, and there were fewer patients with pulmonary hypertension (PH). On the other hand, the inferior vena cava (IVC) was dilated in a lower percentage of patients, with a greater presence of inspiratory collapse ≥ 50%. Regarding the presence of valvulopathies, patients with low CA-125 blood levels presented less mitral regurgitation (MR) and tricuspid regurgitation (TR) (Table 5). Multivariable analysis showed that only an inspiratory collapse of the IVC greater than 50%, and not severe RV dysfunction, was associated with a CA-125 value ≤ 50 U/mL. In the multivariable statistical model, clinically relevant parameters, such as RV diameter, the presence of significant TR, IVC dilatation, and the presence of PH, lost significance (Figure 1).

### 3.4. Survival and Risk Prediction

**Figure 2:**
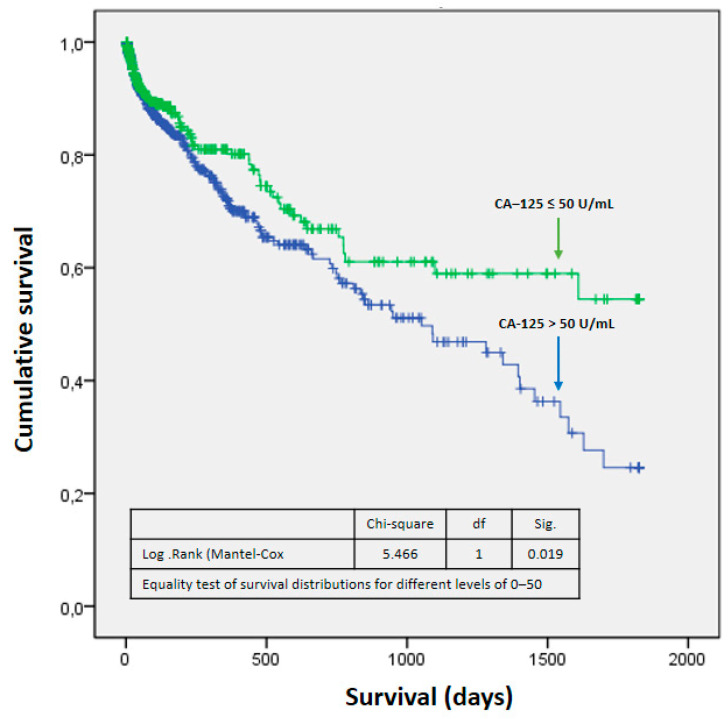
Survival function. Abbreviations: fd—freedom degrees; Sig— statistical significance.

**Figure 3:**
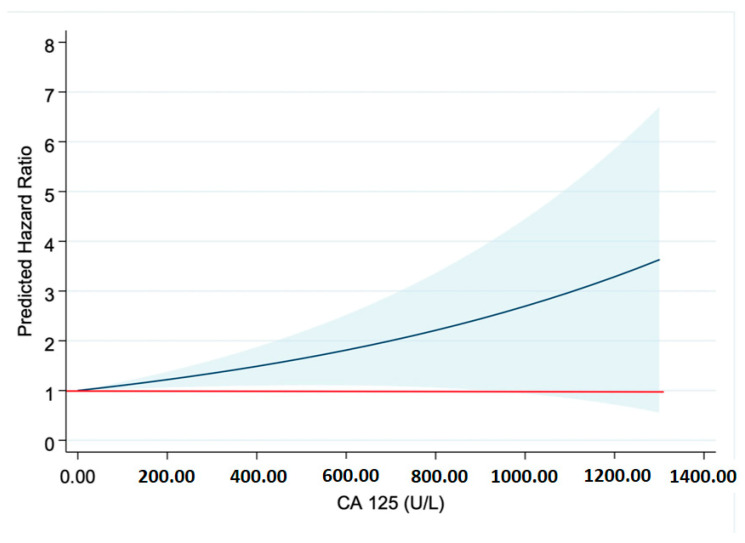
Risk prediction. Abbreviations: CA-125—carbohydrate antigen 125. The red line marks the Hazard Ratio (HR) of 1. The blue line shows the predicted HR for each CA-125 value.

**Figure 4:**
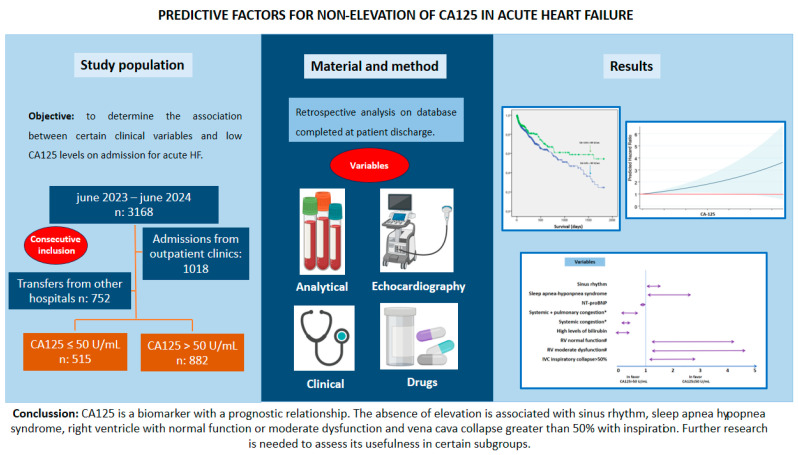
Graphical abstract. Abbreviations: CA-125—carbohydrate antigen 125; HT— hypertension; LV—left ventricle; NT-proBNP—amino-terminal propeptide B-type fragment; RV—right ventricle.

Differences were found in the probability of survival between both groups (*p*: 0.019), such that it was higher in the CA-125 ≤ 50 U/mL group (Figure 2). These differences were more marked from the first year after admission (Table 6). It could be seen that as CA-125 values increased, mortality increased. From a statistical point of view, this was due to greater dispersion and a decrease in expected mortality at values above 800 U/mL (Figure 3). Figure 4 shows a graphical summary of the main results of the analysis.

## 4. Discussion

CA-125 is a relatively recently discovered biomarker used in HF, and it is primarily associated with congestion and prognosis, especially in chronic ambulatory settings [10]. However, it has been less studied in episodes of worsening HF. It is linked to both intravascular and interstitial congestion [1,5,6], and most patients experience an increase in this biomarker during acute cardiac decompensation [10]. Nevertheless, CA-125 levels are not always elevated in patients admitted for congestion, and the scientific literature has yet to clarify why this occurs or what the absence of elevation may indicate. Therefore, at present, CA-125 should be considered an experimental biomarker, requiring further basic and clinical research before its clinical validation and standardization. In this study, certain clinical characteristics were independently associated with the absence of CA-125 elevation, including sinus rhythm, SAHS, low NT-proBNP levels, and inferior vena cava (IVC) collapse >50% with inspiration. Conversely, CA-125 levels above 50 U/mL have been associated with worse prognosis in the medium term (five years). Additionally, an inverse relationship between CA-125 levels and survival probability has been observed.

Of the patients admitted for acute HF, the majority presented elevated CA-125 (63%). This is consistent with the physiology of this biomarker, as mechanical stretch/stress induced by serous fluid accumulation stimulates mesothelial cells, which then produce and increase the release of CA-125 [10]. But, in addition, the inflammatory response is overactivated in HF, which contributes to the overexpression of CA-125 by mesothelial cells [11]. In studies performed in acute HF, the mean CA-125 at discharge is variable, ranging from 38 to 100 U/mL [12,13]; however, no data on the percentage of patients admitted with acute HF and normal CA-125 are found in these articles. This is relevant because it is not clear why congestive patients present non-high levels of this biomarker. Regarding the selected cut-off point (50 U/mL), it was chosen based on the previous literature and its clinical relevance in the context of acute HF. The selected cutoff of 50 U/mL aligns with values commonly used in prior research to differentiate between patients with significant congestion and those with lower biomarker expression. Additionally, this threshold helps to reduce variability by excluding minor elevations that may not be clinically meaningful, ensuring that only cases with a relevant CA-125 increase are considered. It should be taken into account that the maximum value of normality is not clearly defined in HF, the value usually used in ovarian neoplasia being 35 U/mL [14,15]. Only one study analyzes cutoff points in acute HF, estimating a value for normality <23 U/mL; however, this point was drawn from analyzing a retrospective cohort with a validation cohort that only included patients up to 2018, where the medical treatment of HF was not the same as that currently available, and CA-125 levels were not measured at the same time in the two cohorts [16].

It was observed in this series that patients admitted for acute HF with low CA-125 expression on admission were more frequently women, with a higher prevalence of sinus rhythm, obesity, and SAHS, with only sinus rhythm and SAHS being independent predictors of low CA-125. Previous studies have shown that plasma CA-125 is closely related to new-onset atrial fibrillation (AF) [17,18,19]. This is because CA-125 and atrial fibrillation (AF) share common pathophysiological pathways, mainly related to venous congestion, inflammation, and atrial fibrosis. Moreover, AF and HF frequently coexist, and both conditions can lead to systemic venous congestion, which in turn stimulates CA-125 production. It has also been associated with AF recurrence after ablation, with high concentrations of this biomarker serving as an independent predictor of AF recurrence after one year [20]. Thus, the clinical relevance lies in the worse prognosis of AF in patients with elevated CA-125, its higher recurrence rate, and the need to consider that a HF patient in sinus rhythm may have lower CA-125 levels compared to a patient with concurrent AF. The relationship between SAHS and low CA-125 in IC could be influenced by several factors, such as obesity, which is frequently associated with it; cardiac dysfunction patterns linked to SAHS; and even the effect of chronic intermittent hypoxia, which has not been specifically studied in terms of CA-125 production.

Regarding pharmacological treatment, we found a trend toward a lower percentage of patients who were taking MRAs and iSGLT2 and had a low CA-125 level on admission. There are no reported data regarding the relationship between this biomarker and MRA therapy, but there are studies that analyze the effect of iSGTL2. One study evaluated the effect of dapagliflozin treatment on short-term CA-125 levels in patients with HF with reduced LVEF (HFrEF), finding that this drug was associated with a significant reduction in CA-125 [21]. However, these results were found in cases of chronic stable HF. The postulated mechanism is osmotic diuresis, together with a reduction in interstitial fluid [22]. CA-125 antigen has been investigated as a biomarker to guide medical therapy in patients with heart failure, particularly in the context of congestion. CA-125-guided medical therapy has been associated with a significant reduction in the composite endpoints of all-cause mortality and rehospitalization for acute HF at one year, which mainly occurred due to a decrease in the number of heart failure rehospitalizations. However, it is important to note that, while the results are promising, further research is needed to definitively establish the role of CA-125 in guiding HF treatment. Currently, clinical practice guidelines do not routinely recommend the use of CA-125 to guide therapy [13].

We analyzed four patterns of congestion previously described by our group [23,24] and found that all clinical patterns present in patients with decompensated HF—systemic congestion, low output, and mixed congestion—were independent predictors of elevated CA-125 on admission when compared to the pattern of pulmonary congestion. Some authors have proposed CA-125 as a marker of right-sided heart failure [25,26]. In a study by Miñana G. et al., which analyzed 2949 patients admitted for acute HF, five factors were identified as being associated with elevated CA-125—the presence of pleural effusion, the severity of tricuspid regurgitation, age, NT-proBNP levels, and peripheral edema (4.3%)—all of which are typically present in right-dominant HF [27]. The classical theory suggests that NT-proBNP correlates with myocardial stress, being more elevated in left HF phenotypes, whereas CA-125 is more closely related to tissue congestion and, consequently, to right-dominant HF [28]. Regarding analytical values, in this study, we observed that patients with low CA-125 levels had lower bilirubin, transaminase, and NT-proBNP levels, with only low NT-proBNP and bilirubin values being independent predictors. In the case of transaminase levels, considering that they are particularly elevated in right ventricular failure [23,24,29], the association of higher values of these biomarkers with elevated CA-125 levels is justified. No relationship was found between low CA-125 and renal dysfunction in the present series, a finding that aligns with previous evidence demonstrating that CA-125 levels are not influenced by renal function [30].

Regarding the relationship and value of CA-125 relative to other traditional biomarkers, CA-125 has emerged as a complementary biomarker in HF risk stratification [31], especially in the identification of systemic congestion, an aspect less well reflected by traditional markers such as NT-proBNP. While NT-proBNP is widely recognized for its correlation with volume overload and ventricular dysfunction, CA-125 provides additional information via links to inflammatory processes, neurohormonal activation, and myocardial fibrosis. Several studies have shown that the combination of both markers improves the prediction of adverse events, as NT-proBNP reflects intracardiac pressure and myocardial wall stress, whereas CA-125 is more closely associated with peripheral congestion and systemic inflammation. Therefore, the joint assessment of these biomarkers could optimize prognostic stratification and therapeutic decision-making in patients with HF, two biomarkers that provide complementary information [32].

Echocardiographically, patients with CA-125 ≤ 50 U/mL more frequently presented preserved LVEF and related data (such as myocardial hypertrophy and left atrial dilatation), whereas RV function was normal in a higher percentage of patients, with less dilatation of this chamber, fewer patients with PH, less IVC dilatation, and a lower prevalence of MR and TR; as commented, only an IVC collapse > 50% with inspiration showed an association with CA values ≤ 50 U/mL. The relationship between CA-125 levels and echocardiographic parameters has yielded conflicting results in some studies, especially in relation to LV function [33]. In several studies in patients with congestive HF, CA-125 levels did not correlate with LVEF or LV end-diastolic diameter [34,35]. However, Yilmaz et al. found that CA-125 levels correlated positively with pulmonary systolic pressure and negatively with ejection fraction. Furthermore, a decrease in LVEF, RV dilatation, and pericardial effusion were independent predictors of elevated CA-125 levels [25]. Although the relationship between CA-125 and LV function parameters is controversial in different studies, its levels are related to pulmonary artery pressure and right atrial pressure. In the setting of PH, the role of CA-125 remains uncertain [36]. A retrospective study by Zhang Y. et al. including 231 patients with idiopathic pulmonary arterial hypertension and chronic thromboembolic PH showed that CA-125 was associated with functional status, hemodynamics, and prognosis [37]. Regarding the association of the absence of significant TR with low CA-125, we again found a relationship with right HF and systemic and mixed congestion, with both situations being closely related to the presence of TR. In fact, CA-125 is considered an important analytical biomarker for risk stratification in patients with this type of valvular heart disease [38]. Less well known is the relationship of MR with CA-125 levels. An analysis of the BIOSTAT-CHF study, which included more than 1000 patients with significant MR, found that they had increased levels of certain biomarkers, including NT-proBNP (related to myocardial stress and especially to left dysfunction) but also CA-125 [39].

Finally, the impact of this biomarker on survival was greater in the high-CA-125 group, especially after the first year of admission. This coincides with what has been found in the literature, since it is known that CA-125 in acute HF is a useful prognostic factor in both the short and long term [33]. It should be taken into account that high values of this biomarker are associated, as has been mentioned, with patterns of systemic congestion, and this type of pattern has been shown to be associated with worse long-term survival than pulmonary congestion, or even low output [24], and so it is possibly not so much the CA-125 values but the patient profile and pathology that are associated with a worse prognosis.

This is a single-center retrospective study performed on a database completed at patient discharge. Therefore, the main limitation lies in the potential biases inherent to the retrospective nature of the study. No analysis was conducted to determine whether there are differences between the excluded and included patients. We did not analyze other causes that could be elevating this biomarker, although in some cases we consulted gynecology for tumor screening, which was always negative. The value of 50 was chosen to compensate the groups and because there is no recommended value in the literature specific to HF, the established values being related to ovarian neoplasia. It is possible that another value would have allowed a different approach, but we consider that this would have been discovered in the analyses. Additionally, this study did not consider the interobserver variability in echocardiography image interpretation. However, the series is consecutive, which gives reliability to the results, and completed by the team of cardiologists in the Heart Failure Unit. In addition, all data are entered and pre-checked by a single cardiologist also on the team.

In summary, elevated CA-125 levels are usually correlated with a greater severity of HF and worse prognosis due to their link with inflammatory processes and fluid retention. However, some patients have surprisingly low CA-125 levels despite obvious cardiac congestion and deterioration. These cases present a diagnostic and prognostic challenge, as low CA-125 levels may be misinterpreted as indicative of lesser severity. This discordance suggests the need to study underlying factors in these patients, such as variations in inflammatory response, variability in mesothelial response, genetic variability in genes regulating CA-125 synthesis or release, differences in the activation of the autonomic nervous system and the renin–angiotensin–aldosterone system, other comorbidities and/or medications, and, of course, the clinical presentation of HF [40]. Understanding this profile could help to optimize clinical evaluation and therapeutic management in congestive heart failure. Prospective studies on this biomarker are still needed to understand its true clinical scope and the safety of making algorithms based on it. We believe that it is still far from being comparable to other common biomarkers used in the diagnosis, prognosis and treatment of HF, such as natriuretic peptides.

## 5. Conclusions

CA-125, determined on hospital admission for acute cardiac decompensation, is a biomarker with a medium-term prognostic capability. The absence of elevation is associated with the presence of sinus rhythm in the ECG, SAHS, low NT-proBNP levels, and bilirubin, as well as, in the echocardiographic study, with collapse of the IVC greater than 50% with inspiration. The best cut-off point in cardiology with which to identify subgroups of patients at higher risk has yet to be determined. Further research is required in this regard.

## Figures and Tables

**Table 1:** Baseline characteristics of the study population.

	CA-125 ≤ 50 U/mL n: 515	CA-125 > 50 U/mL n: 882	*p*	Total n: 1397
Age (years) #	76 (14)	74 (17)	0.0001	75 (16)
Sex (female)	232 (45)	315 (36)	0.001	547 (39)
Baseline heart disease			0.001	
HT	91 (18)	102 (12)		193 (14)
Ischaemic	145 (28)	260 (29)		405 (29)
IDCM	66 (13)	129 (15)		195 (14)
Valvular	152 (30)	235 (27)		387 (28)
Other	61 (12)	157 (18)		218 (16)
Previous CVS	93 (18)	194 (22)	0.027	287 (21)
HT	427 (83)	662 (75)	0.001	1089 (78)
Dyslipidaemia	319 (62)	530 (60)	0.242	849 (61)
Diabetes mellitus	227 (44)	397 (45)	0.306	624 (45)
Active smoking	187 (37)	380 (43)	0.015	567 (41)
Active drinking	31 (6)	45 (5)	0.422	76 (5)
COPD	82 (16)	124 (14)	0.532	206 (15)
SAHS	93 (18)	88 (10)	0.0001	181 (13)
Obesity	108 (21)	132 (15)	0.001	240 (17)
Renal failure	180 (35)	327 (37)	0.561	507 (36)
Hypothyroidism	57 (11)	71 (8)	0.058	128 (9)
Sinusal rhythm (ECG)	242 (47)	362 (41)	0.028	604 (43)
Stroke	52 (10)	106 (12)	0.625	158 (11)
PVD	57 (11)	88 (10)	0.840	145 (10)

# Kolmogorov–Smirnov 0.0001. Median and interquartile range. Values are expressed as absolute numbers and percentage (in parentheses). Abbreviations: CA-125: carbohydrate antigen 125; COPD: chronic obstructive pulmonary disease; CVS: cardiovascular surgery; IDCM: idiopathic dilated cardiomyopathy; PVD: peripheral vascular disease; HT: hypertension; SAHS: sleep apnea–hypopnea syndrome.

**Table 2:** Clinical profile of patients.

	CA-125 ≤ 50 U/mL n: 515	CA-125 > 50 U/mL n: 882	*p*	Total n: 1397
De novo HF	139 (27)	230 (26)	0.801	369 (26)
Functional class (NYHA)			0.774	
I	72 (14)	115 (13)		187 (13)
II	304 (59)	512 (58)		816 (58)
III, IV	139 (27)	256 (29)		395 (29)
Hemodynamic pattern			0.0001	
Pulmonary congestion	366 (71)	459 (52)		825 (59)
Mixed congestion	88 (17)	238 (27)		326 (23)
Low output	21 (4)	53 (6)		74 (5)
Systemic congestion	41 (8)	132 (15)		173 (13)
Previous admissions	180 (35)	318 (36)	0.642	498 (36)

Values are expressed in absolute numbers and percentage (in parentheses). Abbreviations: CA-125: carbohydrate antigen 125; HF: heart failure; NYHA: New York Heart Association.

**Table 3:** Treatment prior to admission.

	CA-125 ≤ 50 U/mL n: 515	CA-125 > 50 U/mL n: 882	*p*	Total n: 1397
ACEI/ARB/ARNI	62 (12)	106 (12)	0.731	168 (12)
Beta-blockers	304 (59)	530 (60)	0.500	834 (60)
MRA	139 (27)	283 (32)	0.053	422 (30)
SGLT2i	93 (18)	194 (22)	0.076	287 (21)
Vericiguat	10 (2)	4 (1)	0.560	14 (1)
Loop diuretic	330 (64)	574 (65)	0.684	904 (23)
Thiazides	98 (19)	168 (19)	0.887	266 (19)
Tolvaptan	5 (1)	2 (2)	0.058	7 (1)
Acetazolamide	62 (12)	140 (16)	0.644	202 (14)

Abbreviations: ACEI/ARB: angiotensin-converting enzyme inhibitors/angiotensin receptor blockers; ARNI: angiotensin receptor and neprilysin inhibitor; CA-125: carbohydrate antigen 125; MRA: mineralocorticoid receptor antagonist; SGLT2i: sodium–glucose cotransporter 2 inhibitors.

**Table 4:** Blood tests on admission.

	CA-125 ≤ 50 U/mL n: 515	CA-125 > 50 U/mL n: 882	*p*	Total n: 1397
Urea (mg/dL)	53 (41)	56 (43)	0.086	55 (42)
Creatinine (mg/dL)	1.2 (0.7)	1.3 (0.9)	0.247	1.2 (0.8)
Glomerular filtration rate (mL/min/1.73 m^2^)	53 (38)	52 (42)	0.582	52 (40)
Bilirubin (mg/dL)	0.7 (0.6)	0.9 (0.9)	0.0001	0.8 (0.7)
GOT/AST (U/L)	21 (13)	25 (19)	0.0001	23 (17)
GPT/ALT (U/L)	18 (14)	21 (20)	0.0001	20 (17)
usTnT (ng/L)	47 (83)	40 (68)	0.555	41 (47)
NT-proBNP (pg/mL)	4310 (6565)	7200 (8838)	0.0001	5562 (8551)
Sodium (mEq/L)	140 (5)	139 (5)	0.001	139 (6)
Potassium (mEq/L)	4.3 (0.7)	4.3 (0.7)	0.334	4.3 (0.7)
Hemoglobin (g/dL)	13.1 (2.6)	12.2 (3.3)	0.279	12.4 (3.2)
Hematocrit (%)	41 (9)	39 (11)	0.463	39 (10)
Platelets (µL, ÷100)	206 (74)	219 (113)	0.053	214 (98)
Uric acid (mg/dL)	7.5 (2.6)	7.2 (4.0)	0.013	7.6 (3.2)
TSAT (%)	20 (12)	16 (11)	0.151	19 (13)
Ferritin (ng/mL)	154 (249)	165 (204)	0.001	170 (262)

In all the variablesKolmogorov–Smirnov 0.0001. Median and interquartile range. Abbreviations: ALT (GPT), alanine aminotransferase; AST (GOT), aspartate aminotransferase; CA-125, carbohydrate antigen 125; NT-proBNP, N-terminal pro-B-type natriuretic peptide; TSAT, transferrin saturation; usTnT, ultrasensitive troponin.

**Table 5:** Echocardiographic evaluation.

	CA-125 ≤ 50 U/mL n: 515	CA-125 > 50 U/mL n: 882	*p*	Total n: 1397
Preserved LVEF (≥50%)	251 (49)	331 (38)	0.0001	582 (42)
Dilated LV (LV-EDD > 56 mm)	149 (29)	397 (45)	0.0001	549 (39)
LVH (>12 mm)	354 (69)	529 (60)	0.001	883 (63)
Severe left atrial dilatation (≥50 mm)	232 (45)	291 (33)	0.032	523 (37)
Significant valvulopathies *				
AoR	30 (6)	54 (6)	0.906	84 (6)
AoS	74 (14)	88 (10)	0.021	162 (12)
MR	76 (15)	206 (23)	0.0001	282 (20)
MS	2 (0.4)	15 (2)	0.064	17 (1)
TR	68 (13)	204 (23)	0.0001	272 (19)
RV function (TAPSE)			0.0001	
Normal	384 (75)	516 (58)		900 (64)
Mild dysfunction	64 (12)	104 (12)		168 (12)
Moderate dysfunction	41 (8)	158 (18)		199 (14)
Severe dysfunction	26 (5)	105 (12)		131 (10)
Dilated RV (Basal diameter > 40 mm)	149 (29)	353 (40)	0.0001	502 (36)
Inferior vena cava (mm) #	19 (4)	22 (4)	0.0001	20 (4)
Vena cava collapse ≥ 50%	360 (70)	406 (46)	0.0001	766 (55)
PH (PAsP ≥ 50 mmHg)	242 (47)	486 (55)	0.024	728 (52)

* moderate–severe + severe. # Kolmogorov–Smirnov 0.0001. Median and interquartile range. Abbreviations: AoR—aortic regurgitation; AoS—aortic stenosis; CA-125—carbohydrate antigen 125; LV-EDD—left ventricular end-diastolic diameter; LVEF—left ventricular ejection fraction; LVH—left ventricular hypertrophy; MR—mitral regurgitation; MS—mitral stenosis; PAsP—pulmonary artery systolic pressure; PH—pulmonary hypertension; RV—right ventricle; TAPSE—tricuspid annular plane systolic excursion; TR: tricuspid regurgitation.

**Table 6:** Probability of survival at follow-up.

	CA-125 ≤ 50 U/mL n: 515	CA-125 > 50 U/mL n: 882	*p*	Global n: 1397
30 días	99%	99%	0.986	99%
1 año	84%	77%	0.002	80%
2 años	69%	66%	0.264	67%
3 años	62%	52%	0.0001	56%
4 años	60%	42%	0.0001	49%
5 años	55%	30%	0.0001	40%

Abbreviations: CA-125—carbohydrate antigen 125.

## Data Availability

Dataset available on request from the authors.
